# The Control of Hyperglycemia by Estriol and Progesterone in Alloxan induced Type I Diabetes Mellitus Mice Model through Hepatic Insulin Synthesis

**Published:** 2014-03

**Authors:** Suman Bhattacharya, Sarbashri Bank, Smarajit Maiti, Asru K. Sinha

**Affiliations:** 1 Sinha Institute of Medical Science and Technology, India;; 2 Department of Biochemistry, Vidyasagar University, Midnapore, India

**Keywords:** Dermcidin isoform 2, Hepatocytes, Menopause, Type 1 diabetes mellitus, Type 1B diabetes mellitus

## Abstract

As much as 20% of the women in menopause are reported to develop type I diabetes mellitus. The cessation of the ovarian syntheses of the female sex hormones is known to cause menopause in women, and the roles of estriol (one of the most abundant estrogens) and progesterone were investigated for hepatic insulin synthesis through estriol and progesterone induced synthesis of nitric oxide in the liver cells. Type 1 Diabetic mellitus mice were prepared by alloxan treatment, Nitric oxide was determined by methemoglobin method. Insulin was determined by enzyme linked immunosorbant assay. Injection of either 3.5 µM estriol or 3.5 nM progesterone to the diabetic mice which cannot synthesize pancreatic insulin, reduced the blood glucose level from 600 mg/dl to 120 mg/dl and 500 ± 25 mg/dl to 120 ± 6 mg/dl in 6 and 10 h respectively with simultaneous increase of the plasma insulin from 0 µunits/ml to 40 µunits/ml and 0 µunits/ml to 9.5 µunits/ml in the case of estriol and progesterone respectively with stimulated NO synthesis. The inhibition of the steroids induced NO synthesis by using NAME (NG-methyl-l-arginine acetate ester) in the reaction mixture resulted in the inhibition of hepatic insulin synthesis. Use of pure NO solution in 0.9% NaCl instead of either estriol or progesterone in the reaction mixture was found to stimulate the hepatic insulin synthesis. Both estriol and progesterone might be involved in the prevention of type 1 diabetes mellitus through the hepatic insulin synthesis even when the pancreatic insulin synthesis was impaired.

## INTRODUCTION

The occurrence of type I diabetes mellitus, (T1DM) which is known to be caused by the destruction of pancreatic β cells (1), is reported to be a common chronic disease in post-menopausal women (2). Prevalence of diabetes mellitus (types 1 and 2) in post-menopausal women is about 10-20% according to age (3). In contrast, the premenopausal women are generally resistant to the diabetogenic assault when compared to the post-menopausic counterpart (4, 5). As menopause is known to be the consequence of the permanent cessation of the ovarian production of the estrogens and progesterones, it might be implied that these well known female sex hormones were involved in the control of T1DM in premenopausal women. However the role of these steroids, if any, particularly in the control of hyperglycemia in the post-menopausal T1DM remains obscure.

The type I diabetes mellitus as mentioned above is however subdivided in 2 categories: (1) Type1A diabetes mellitus (T1ADM) and (2) Type1B diabetes mellitus (T1BDM) (6). The T1ADM is reported to be the consequences of the destruction of the pancreatic β cells by auto immunologic assault (7, 8). The T1BDM, on the other hand, has been reported to develop due to stresses induced by environmental factors (9). We have recently reported the appearance of a stress induced protein of MW.11,000 kDa identified to be dermcidin isoform 2 (dermcidin) in the circulation of the subjects with T1BDM (10). The stress induced protein itself was found to be capable of inducing T1BDM both in humans (11) and in animal model (12). Furthermore it has also been reported that T1BDM occurred as a major form of T1DM in many parts of the world due to the dermcidin induced inhibition of glucose uptake, rather than to the destruction of the pancreatic β cells, where glucose has an essential role both in the synthesis and secretion of insulin for the control of hyperglycemia (13, 14) both in the pancreatic β cells (13) as well as in the hepatocytes in the liver (15).

We have also reported before that although the pancreatic β cells were capable of synthesizing and secreting insulin when stimulated by glucose, the hepatic cells in adult mice were also capable of synthesizing and secreting insulin in the presence of the sugar (15). It was also found unlike in the case of T1ADM, that the alloxan induced T1DM in the mice did not simultaneously impair the capability of the hepatic cells to express of both proinsulin genes I and II where the gene products were converted to bioactive insulin (10). However, it was also found that normal liver cells did not produce insulin when stimulated by glucose alone, but for the synthesis of insulin in the liver cells, the simultaneous presence of both NO and the sugar were needed (12). The inability of the sugar by itself to induce insulin synthesis and secretion in the hepatocytes was found to be due to the presence of dermcidin (12), a potent inhibitor of NO synthesis (16) and the stimulation of NO synthesis was found to overcome the inhibitory effect of dermcidin in insulin synthesis (12). It was also reported before that the hyperglycemia in T1BDM in humans developed due to the presence of dermcidin could be controlled by stimulating the systemic NO synthesis without using external insulin administration (10).

In the context of the systemic stimulation of NO synthesis, we have described first time ever, that estradiol, estriol and progesterone the well known female sex hormones, were capable of stimulating NO synthesis through the activation of the hormone receptors in both the anucleated (17) and in the nucleated cells (18). Where the receptors themselves were involved in the transportation of the steroids through the lipid membrane bilayer instead of currently held belief that these lipid soluble compounds freely passed through the lipid membrane bilayer (19), and more importantly the effect of these hormones were mediated through the synthesis of NO which when present in the incubation mixture were capable of producing the effect of the hormones themselves through *de novo* protein synthesis in the absence of the added hormones in the reaction mixture (18, 20).

As 20% of the menopausic women are reported to develop T1DM (3), and as the occurrence of menopause in women resulted in the permanent cessation of ovarian synthesis of both estrogens and progesterons, investigations were carried out to determine the role of both estrogens and progesterons on the insulin synthesis through the stimulation of NO synthesis in the liver cells in alloxan treated mice where the pancreatic β cells were destroyed which consequently resulted in overt hyperglycemia due to the inhibition of Insulin synthesis.

## METHODS

### Ethical Clearance

This study used Swiss white mice as an animal model for T1DM induced by alloxan (21). Appropriate permission was obtained from the Internal Review Board, Sinha Institute of Medical Science and Technology, Calcutta.

### Chemicals

Estradiol, estriol and progesterone, the Goat anti-rabbit immunoglobulin G-alkaline phosphatase were obtained from Sigma Aldrich. ELISA Maxisorb plates were from Nunc, Roskilde, Denmark. All other chemicals used were of analytical grade. These hormones were solubilised in 0.9% NaCl.

### Preparation of alloxan induced mice

Normal healthy mice 2-3 month old (25-30 gm weight) were used to develop diabetic mice by the injection of alloxan as described before (21). Before these animals were treated with alloxan, only those mice that had ≈ 120 mg glucose/dl after overnight “fasting” were consider to be “non diabetic” mice and used for further studies. These animals were inbred animals and they were raised in our animal facility from birth. They were fed standard laboratory chow, and sterilized water was given *ad libitum.* The animals were grown in 12 h cycles of light and darkness at 23°C. Before use these mice were checked by a licensed veterinarian to determine that these animals were free from diseases.

The preparation of diabetic mice by using streptozotocin, as an alternative T1DM mice model, was not attempted due to several reports that demonstrated streptozotocin was both carcinogenic and mutagenic antibiotic can seriously damage liver in mice (22, 23). As in our intended study it was essential that liver cells were not damaged for the glucose induced synthesis of insulin in the hepatic cells (15), the use of streptozotocin induced diabetic mice in our study was not followed up. On the other hand, it has also been reported that alloxan did not damage liver in mice (24).

### Preparation of mice liver cell homogenate

Adult mice were killed by cervical dislocation and the entire liver was immediately excised out and placed in cold (0°C) Tyrods buffer (pH 7.4) and the homogenate of the excised liver was made in the same buffer as described before (25).

### Assay of estrogens and progesterones induced NO synthesis in the liver cells homogenate and the determination of NO and glucose in the mice blood

Typically, 0.1ml of the liver cell homogenate in Tyrods buffer (pH 7.4) was incubated with different amounts of estriol, estradiol or progesterone in the presence of 2.0 mM CaCl_2_ at 37°C in the presence of 1.0 µM *l-arginine* in a total volume 1.0 ml for 30 min. The synthesis of NO was determined in the cell free reaction mixture supernatant by methemoglobin method by determining the spectral changes of absorption maxima at 525 and 630 nm under N_2_ by using a Beckman Spectrophotometer as described before (26). The quantitation of NO was independently verified by chemiluminescence method (27).

The plasma level of NO in mice was determined by similar method by withdrawing 0.2 ml of blood from the tail vein of mice.

The blood glucose level was determined by using a glucometer (Behring).

### Determination of estriol, estradiol induced synthesis of insulin

The plasma level of insulin in mice was determined by enzyme linked immunosorbant assay (ELISA) by using anti insulin antibody as described in details before (28). For this purpose the blood sample (0.1 ml) was drawn from the tail vein of mice.

### Determination of synthesis of insulin

In some of the experiments it was necessary to determine the synthesis of insulin in the reaction mixture. The synthesis of the hormone was determined by in vitro insulin mRNA synthesis and quantitated by ELISA as described above.

### Statistical Methods

Results shown are mean ± standard deviation (S.D) of the experiments each in triplicate by using at least 3 different animals. The significance of the results was determined by student’s t- test. The significance “*P*”<0.001 was considered to be significant. Correlation coefficient, Pearson score “*r*”, is such that -1 ≤ r ≤+1 is acceptable. The (+) and (-) signs are used for positive linear correlations and negative linear correlations, respectively.

## RESULTS

### Effect of administration of physiologic amounts of estriol on the blood glucose and NO levels in alloxan induced mice

**Figure 1:**
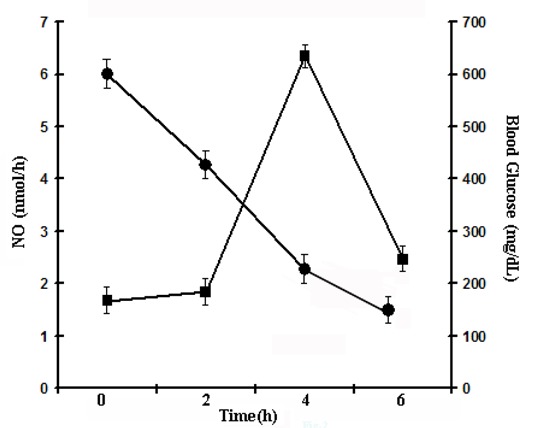
Time course of estriol induced increase of plasma nitric oxide level and the reduction of the blood glucose level in alloxan induced diabetic mice. Each point mean ± S.D. of each experiment in triplicate by using 5 different animals injected with 3.5 µM estriol. Solid circles (●) represent blood glucose and solid squares (■) show nitric oxide level.

It was reported before that estriol, one of the most abundant estrogens in women (29), was a potent activator of NO synthesis in nucleated cells due to the activation of the hormone receptors on the cells (18). To determine the effect of estriol on the blood sugar and NO levels in alloxan treated mice, if any, when the estrogen at physiologic concentration (3.5 μM) was injected in the circulation of the test animal, it was found that the blood NO level began to increase after 2 h of the administration of the hormone, and was maximally increased at 4 h. Subsequently, the increased blood NO level was again found to decrease (Fig. 1). When the blood glucose levels were determined at different times in the same animal it was found the initial blood sugar level of >600 mg/dl in the alloxan treated mice decrease to 120 mg/dl at 6 h. However it was noted that the decrease of the blood glucose level was somewhat lagged behind the increase of the blood NO level *in vivo* in these animal. The correlation coefficient (“r”) between the increase of plasma NO level and the decrease of plasma glucose level was -0.945 indicating the increase of the plasma NO level was inversely related to the decrease of the plasma glucose level.

**Figure 2:**
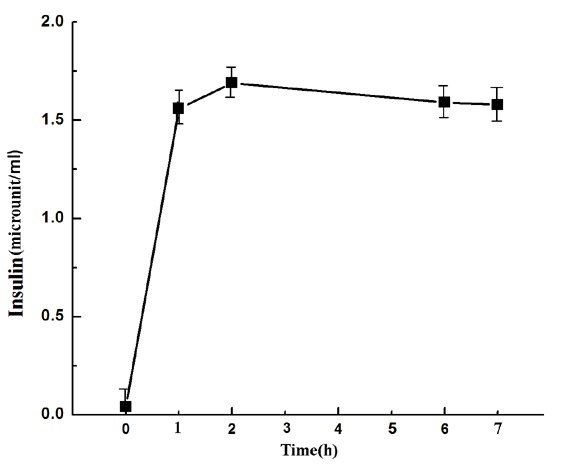
Estriol induced synthesis of insulin in alloxan treated mice at different time. The alloxan treated diabetic mice was injected with 3.5 µM estriol and the plasma insulin level was determined by enzyme linked immunosorbant assay as described in the Methods. Each point was mean ± S.D. at least 10 different experiments using 10 different alloxan treated diabetic mice.

As the decrease of the blood glucose level in alloxan induced mice where the pancreatic insulin synthesis was not possible, the estriol induced marked decrease of the blood glucose level in the alloxan treated mice implied an extra pancreatic synthesis of insulin for the control of hyperglycemia. When the plasma insulin level in the same estriol treated mice was determined, it was found that the plasma hormone level of ≈ 0 µunits/ml before the treatment of the mice with estriol increased to 40 µunits of insulin/ml (Fig. 2) at 6 h.

### Effect of estriol on the synthesis of NO and insulin in the liver cell homogenate

**Figure 3:**
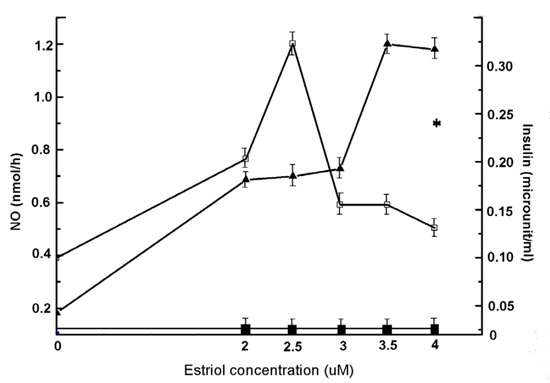
Effect of treatment of liver homogenate from normal mice with different concentrations of estriol on the synthesis of NO and insulin. The liver homogenate was prepared from the liver of adult mice as described in the Methods. The liver homogenate was treated with different amounts of estriol as indicated. After incubation at 37°C for 30 min synthesis of both NO and insulin were determined. Results shown are mean ± S.D. of at least 6 different experiments each in triplicate using 6 different animals. While the solid triangles (▲) represent NO synthesis and hollow squares (□) show insulin synthesis. Solid squares (■) show the estriol induced insulin synthesis in the liver reaction mixture in the presence of both estriol and 1 mM NAME. And (*) represents the synthesis of insulin in presence of 2.5 nmol NO only.

Although the results described under Fig. 1 and Fig. 2 demonstrated the synthesis of insulin could be related to the estriol induced NO synthesis in the whole animal, these results did not specify that source of estriol induced synthesis of either insulin or NO or whether the synthesis of insulin was actually related to NO synthesis. As reported before the liver cells from alloxan treated mice were capable of synthesizing insulin (12) to determine the role of the hepatic insulin synthesis in the control of hyperglycemia in alloxan treated mice, when the liver cell homogenate was prepared as described in the Methods, and treated with different concentrations of estriol, it was found the maximal NO was achieved at 3.5 µM (Fig. 3). When the synthesis of insulin was determined in the same reaction mixture, it was found that the synthesis of insulin, as determined by *in vitro* translation of insulin mRNA in the liver cell homogenate in the presence of estriol, it found that the estriol treated liver cell homogenate synthesized insulin ≈ 9 µunit/ml (Fig. 3). These results indicated that the liver was indeed the source of estriol induced insulin and NO syntheses. The treatment of the liver cell homogenate with 1mM NAME (N^G^-methyl-l-arginine acetate ester), an inhibitor of NOS [30], not only inhibited the estriol induced NO synthesis but simultaneously nullified the estriol induced insulin synthesis in the liver cell homogenate (Fig. 3). It was also found that instead of adding estriol to the incubation mixture direct addition of NO solution in 0.9% NaCl was also capable of synthesizing insulin in the reaction mixture. (n= 6, *p*<0.005).

### The effect of estradiol on the reduction of hyperglycemia in alloxan treated mice

**Figure 4:**
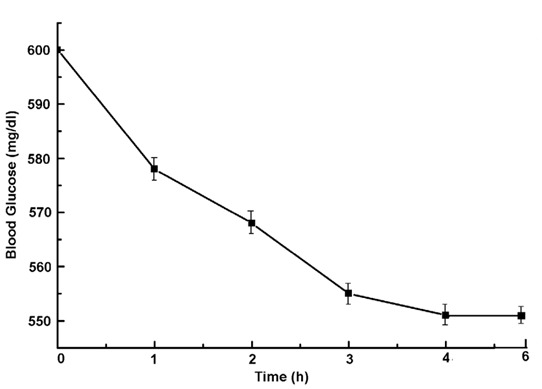
Effect of injection of estradiol on the reduction of the blood glucose level in alloxan induced diabetic mice. Estradiol (3.5 µM) was injected in the circulation of the alloxan treated mice and the blood glucose level was determined at different time after injection of estradiol as indicated. Results shown are mean ± S.D. of 5 different experiments each in triplicate by using 5 different alloxan induced diabetic mice.

Unlike estriol when estradiol, the most potent estrogenic hormone, was injected in the diabetic mice, it was found that use of 3.5 µM estradiol instead of the similar amount of estriol was found to decrease the blood glucose level much less effectively (≈550 ± 25 mg/dl in the diabetic mice reduced the blood glucose level only to 410 ± 20.5 mg/dl). (Fig. 4) (n=5, *p*<0.0001).

### The effect of progesterone on synthesis of insulin and nitric oxide in the mice liver homogenate

**Figure 5:**
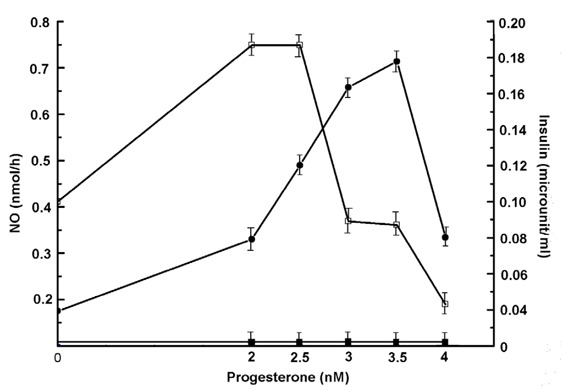
Effect of progesterone on the synthesis of NO and insulin in the liver homogenate from the normal mice. Liver homogenate from normal adult mice was prepared in Tyrods buffer (pH 7.4) as described in the Methods. Typically 0.1 ml of the homogenate was incubated with different amounts of progesterone as indicated in the presence of 2.0mM CaCl2 and 1.0 µM *l-arginine* in a total volume of 1.0 ml. After incubation for 30 min at 37°C, both the synthesis of NO and insulin were determined. Results shown are mean ± S.D. of experiment each in triplicate by using 5 different animals. Solid circles (●) and hollow squares (□) indicate NO and insulin synthesis respectively. The solid squares (■) represent the addition of NAME (1mM) to the reaction mixture on the synthesis of both NO and insulin.

As we have reported before that progesterone was a more potent steroid than estradiol in the synthesis of NO (20), the effect of progesterone on the hyperglycemia in the alloxan treated mice were determined. To determine the effect of progesterone, also an ovarian steroid, when the liver cell homogenate was treated with different concentrations of progesterone it was found that progesterone at nM concentrations was able to increase both NO and insulin synthesis in the liver cell homogenate (Fig. 5). The inhibition of the progesterone induced NO synthesis by NAME as in the case of estriol resulted in the nullification of both NO and insulin synthesis. (n=5, *p*<0.005).

These results demonstrated that progesterone on weight/weight (nM vs μM) was a thousand fold more effective stimulator of insulin synthesis in the liver cell homogenate than that of estriol.

### Effect of progesterone on the control of hyperglycemia through the stimulation of insulin in alloxan treated mice

**Figure 6:**
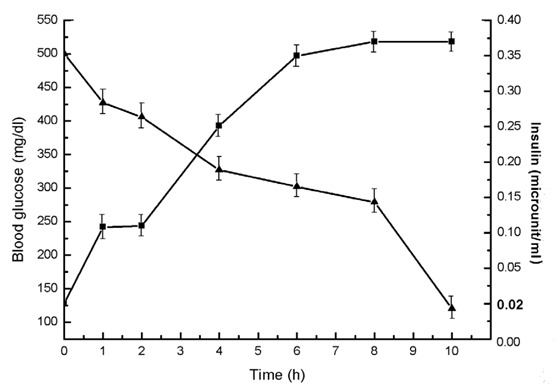
The effect of injection of 3.5 nM (Final) progesterone in the circulation of alloxan induced diabetic mice on the blood glucose and insulin level *in vivo*. Alloxan induced diabetic mice were prepared as described in the Methods. Progesterone (3.5 nM) was injected in the circulation of the test animals. Each point represents mean ± S.D. of each experiment in triplicate by using 5 different diabetic mice at different time as shown. Solid triangles (▲) and solid squares (■) represent blood glucose and plasma insulin levels respectively.

As the above results indicated that the treatment of the liver cell homogenate from the alloxan treated mice with nM amounts of progesterone was capable of inducing insulin synthesis through the stimulation of NO synthesis in the liver cell preparation, the effect of injection of progesterone in alloxan induced diabetic mice was determined. It was found that the injection of 3.5 nM (final) progesterone in the circulation of the diabetic mice resulted in the reduction of the initial blood glucose level of 500 ± 25 mg glucose/dl to 120 ± 6 mg glucose/dl after 10 h of the injection (Fig. 6).

Determination of the plasma insulin levels at different times in the same alloxan treated mice that received 3.5 nM progesterone demonstrated to increase of plasma insulin level of ≈ 0 μ units/ml before the injection of the hormone increase to 9.5 µunits/ml after 4 h to at least 8 h after the injection of the ovarian steroid (Fig. 6) (n=5, *p*<0.0001).

### Dose response of the progesterone induced reduction of hyperglycemia in the alloxan induced diabetic mice

As described under the Fig. 6, progesterone at 3.5 nM in the alloxan induced diabetic mice could control the overt hyperglycemia of 500 ± 25 mg/dl to 120 ± 6 mg/dl in 10 h. To determine the minimal amount of progesterone capable of controlling hyperglycemia in alloxan treated mice, when different amounts of the steroid were injected into the test animal it was found that as little as 1 nM progesterone was capable of reduction the blood glucose level of 300 ± 15 mg/dl to 170 ± 8.25 mg/dl in the diabetic mice in the same time (n=5, *p*< 0.001).

## DISCUSSION

The occurrence of type 1 diabetes mellitus (T1DM) in ≈ 20% of the post-menopausic women is a serious health problem among these victims. Although no mechanism is currently available for the development of T1DM in these subjects, the condition nevertheless is reported to be a major risk factor for the development of atherosclerosis (31). Fallaciously however, the mechanism of development of atherosclerosis itself remains obscure. The development of atherosclerosis is known to cause prothrombotic condition that result in the life threatening acute ischemic heart disease (AIHD) (32). Among the post-menopausal women the contributory role of T1DM developed in the post-menopausal women as an atherosclerotic risk factor leading to the life threatening AIHD in these victims cannot be ruled out.

The genesis of T1DM in post menopausal women could however, be related to the permanent cessation of the syntheses of female sex hormones in the ovaries which, in the first place, caused menopause in the victims. The results presented above indicated that the cessation of ovarian hormones might actually be involved, at least partly, in the genesis of T1DM in women due to menopause. This conclusion was drawn from the results that demonstrated the use of both estrogen (particularly estriol) and progesterone in alloxan induced T1DM in the animal model effectively controlled hyperglycemia through the hepatic synthesis of insulin due to the stimulation of NO synthesis mediated through the activation of the estrogen or progesterone receptors on the cell surface as reported before (18, 20). In this respect, on weight/weight basis, progesterone was found to be a thousand fold more effective hypoglycemic agent than estriol *in vivo* through the synthesis of insulin in the liver cells due to NO synthesis (Fig. 5 and Fig. 6). Although progesterone is implicated in the etiology of the gestational diabetes and as such, our results might however contradict the effect of progesterone on the control of hyperglycemia, it should be mentioned that the gestational diabetes is not caused by a single reason. In that obesity, hyperinsulinemia, insulin resistant diabetes mellitus that is type 2 diabetes mellitus along with metabolic stresses are involved in the gestational diabetes and as such, the effect of progesterone in the control of hyperglycemia in gestational diabetes might not be feasible. The ability of either estriol or progesterone to initiate insulin synthesis was apparently mediated by neutralization of the dermcidin isoform 2 (11) induced inhibition of glucose uptake in the pancreatic β cells for the synthesis of insulin that we have reported before (11).

As the stimulation of NO synthesis by either estriol or progesterone resulted in the control of hyperglycemia through insulin synthesis, it could be inferred that T1DM was not only caused by the destruction of pancreatic β cells, but a T1BDM like condition due to environmental stress was also present in these animals due to the systemic presence of dermcidin (10), a potent inhibitor of all known forms of nitric oxide synthases (16). The effect of NO in relation to hypoglycaemia has never been reported before due to the neutralization of dermcidin isoform 2, a universal inhibitor of nitric oxide synthases via the hepatic insulin synthesis. In this context it should be mentioned here that it was not estradiol, the most potent estrogenic hormone, but estriol, an abundant estrogen but less potent than estradiol as an estrogenic hormone in the females, itself was more efficacious in the control of hyperglycemia through hepatic insulin synthesis in the alloxan induced T1DM mice, and progesterone, however, was found to be the most potent antidiabetic agent in these diabetic mice in the above context. In hormone replacement therapy it was estradiol but not estriol is used and as such, as we have described in the manuscript estradiol itself did not have impressive effects in the control of hyperglycemia through hepatic insulin synthesis. However, hormone replacement therapy has been reported to control diabetes mellitus (32).

As both estriol and progesterone were capable of controlling hyperglycemia in the alloxan induced mice model for T1DM, and as the pancreatic β cells were destroyed by the diabetogenic compound, the anti diabetic effect of these steroids cannot be mediated through the pancreatic synthesis of insulin, an essential hormone in glucose metabolism, and the steroids were capable of inducing insulin synthesis in the liver cells not only *in vivo*, but both estriol and progesterone was found to be efficiently synthesized insulin due to the steroids induced NO synthesis *in vitro* liver homogenate as well. The inhibition of NO synthesis by NAME resulted in the inhibition of the syntheses of both NO and insulin in the liver homogenate from normal adult mice. These results supported our earlier claim that mammalian hepatic cells were capable of synthesizing insulin and suggested that the systemic presence of functional hepatocytes was capable of synthesizing insulin even where the pancreatic β cells were destroyed.

Finally, although ≈ 20% of all post-menopausic women are reported to develop T1DM, but the percent of T1ADM and T1BDM in these subject are not known. And, as such many of these T1BDM victims in the post-menopausic women could be T1BDM caused by the environmental stresses including the stresses produced by the cessation of the synthesis of the ovarian hormones, believed to be one of the most stressful events in the lives of women in general.

If the results obtained from the animal model of T1DM presented above could be extended to the human conditions, particularly due to the fact that there is no report is available to suggest that the cessation of ovarian hormone synthesis resulted simultaneously in the destruction of hepatocytes in the liver in these subjects. And, as such, the use of nM (final) amounts of progesterone might be useful for the control of T1BDM in the post menopausic women through hepatic insulin synthesis to prevent AIHD.

In this context it may be mentioned here that post prandial plasma levels of insulin are known to be in the sub nM ranges for the control of hyperglycemia in humans, and as reported above progesterone was also found to be capable of controlling hyperglycemia also at nM ranges in alloxan induced diabetic mice through the hepatic insulin synthesis (Fig. 6).

## CONCLUSION

It could be concluded that ovarian hormones including estrogen and progesterone particularly might have a significant role in the control of hyperglycemia at least in the animal where the pancreatic β cells are dysfunctioned, through the production of insulin due to the estrogen and progesterone receptor activated NO synthesis in the liver cells. It is also concluded that estriol or progesterone could be of value in control hyperglycemia in post-menopausal women who are known to develop T1DM at higher rate compare to appropriate control.

## ABBREVIATIONS

T1DM: Type 1 Diabetes mellitus
T1ADM: Type 1A Diabetes mellitus
T1BDM: Type 1B Diabetes mellitus
NAME: N^G^-methyl-l-arginine acetate ester
